# Multifunctional Ablative Gastrointestinal Imaging Capsule (MAGIC) for Esophagus Surveillance and Interventions

**DOI:** 10.34133/bmef.0041

**Published:** 2024-04-03

**Authors:** Hyeon-Cheol Park, Dawei Li, Rongguang Liang, Gina Adrales, Xingde Li

**Affiliations:** ^1^ Department of Biomedical Engineering, Johns Hopkins University, Baltimore, MD 21205, USA.; ^2^ Department of College of Future Technology, Peking University, Beijing, 100871, China.; ^3^ College of Optical Sciences, University of Arizona, Tucson, AZ 85721, USA.; ^4^ Department of Surgery, Johns Hopkins University School of Medicine, Baltimore, MD 21205, USA.

## Abstract

**Objective and Impact Statement:** A clinically viable technology for comprehensive esophagus surveillance and potential treatment is lacking. Here, we report a novel multifunctional ablative gastrointestinal imaging capsule (MAGIC) technology platform to address this clinical need. The MAGIC technology could also facilitate the clinical translation and adoption of the tethered capsule endomicroscopy (TCE) technology. **Introduction:** Recently developed optical coherence tomography (OCT) TCE technologies have shown a promising potential for surveillance of Barrett’s esophagus and esophageal cancer in awake patients without the need for sedation. However, it remains challenging with the current TCE technology for detecting early lesions and clinical adoption due to its suboptimal resolution, imaging contrast, and lack of visual guidance during imaging. **Methods:** Our technology reported here integrates dual-wavelength OCT imaging (operating at 800 and 1300 nm), an ultracompact endoscope camera, and an ablation laser, aiming to enable comprehensive surveillance, guidance, and potential ablative treatment of the esophagus. **Results:** The MAGIC has been successfully developed with its multimodality imaging and ablation capabilities demonstrated by imaging swine esophagus ex vivo and in vivo. The 800-nm OCT imaging offers exceptional resolution and contrast for the superficial layers, well suited for detecting subtle changes associated with early neoplasia. The 1300-nm OCT imaging provides deeper penetration, essential for assessing lesion invasion. The built-in miniature camera affords a conventional endoscopic view for assisting capsule deployment and laser ablation. **Conclusion:** By offering complementary and clinically viable functions in a single device, the reported technology represents an effective solution for endoscopic screening, diagnosis, and potential ablation treatment of the esophagus of a patient in an office setting.

## Introduction

Over the past few decades, endoscopic optical coherence tomography (eOCT) has shown great potential for early diagnosis of esophageal cancer [1–3]. One of the most salient examples is using OCT to augment the detection of dysplasia during Barrett’s esophagus (BE) surveillance, the established precursor to esophageal adenocarcinoma (EAC) [4,5]. Periodic screening and detecting the dysplasia associated with BE are crucial for monitoring the progression from BE to EAC and enabling timely interventions [5,6]. In this regard, eOCT has long been regarded as a promising imaging technology for detecting the dysplasia associated with the BE owing to its capability of real-time cross-sectional imaging of mucosal and submucosal layers with micron-scale resolution [7–10]. Moreover, eOCT may greatly reduce errors associated with random biopsies that rely on conventional endoscope camera images of superficial mucosa [11,12].

The first generation of eOCT catheters for esophagus imaging utilized flexible fiber and micro-optics integrated inside an inflatable balloon [13–17]. These fiber-optic catheters can be deployed through an accessory port of a standard gastrointestinal (GI) endoscope during a routine esophagogastroduodenoscopy procedure, enabling comprehensive volumetric imaging of the esophagus by helical scanning of the optics inside a balloon. To ensure reliable imaging and optimal placement of the catheter, the balloon could be inflated until it fully contacts the surface of the esophagus. However, esophagogastroduodenoscopy requires patients to be sedated in specialized settings with special equipment and continuous monitoring of potential adverse reactions by trained medical professionals. It is time-consuming and costly and would not be preferable for regular surveillance nor suitable for general screening purposes [18].

The recently developed OCT-tethered capsule endomicroscopy (TCE) technology overcomes some of the challenges mentioned above. It does not require guidance from a standard endoscope and thus can eliminate the need for patient sedation [19–22]. Similar to wireless capsule endoscopy, the tethered capsule can be swallowed and well tolerated by unsedated patients. Once swallowed, the capsule can acquire volumetric OCT images as it naturally progresses through the digestive system via gravity and peristalsis. Alternatively, the capsule can be pulled upwards toward the mouth using the tether, allowing for comprehensive imaging throughout the area it traverses. It is more patient-friendly and holds great promise for efficient and comfortable GI surveillance, as it eliminates the need for sedation and can be conducted by nurses or technicians even in primary care clinics [23,24].

Conventional eOCT devices utilize a broadband light source with a center wavelength of around 1300 nm, affording an axial resolution of ~7 to 20 μm and a few millimeters of the imaging depth in tissue. These systems successfully identified microarchitectural features associated with neoplasia in BE, such as irregular glandular morphology and increased surface signal intensity, achieving a sensitivity and specificity of 70 to 80% [25,26]. With the current TCE technology, it remains challenging to detect early lesions mainly due to its suboptimal resolution and imaging contrast for OCT operating at 1300 nm [25–27]. The recently developed ultrahigh-resolution (UHR) OCT endoscope and capsule technologies employing a broadband light source around 800 nm exhibit much improved axial resolution (~2.8 μm in air corresponding to ~2 μm in tissue) as well as enhanced imaging contrast owing to stronger light-tissue interactions around 800 nm than 1300 nm [22,28–31]. It has been shown that the UHR OCT capsule is more effective for detecting features associated with esophageal neoplasia [31]. On the other hand, the UHR OCT system compromises the imaging depth to less than 1 mm and may be restrictive in some applications, such as evaluating the invasion depth of squamous cell carcinoma [32] or EAC [33] where a sufficient imaging depth is crucial.

Despite the promising potential of the TCE technology, its adoption by clinicians faces several challenges. One major hurdle is the substantial difference between OCT images and conventional endoscope camera images. The unique characteristics and features captured by OCT may be unfamiliar to many clinicians and would require extensive training in order to interpret and analyze OCT images effectively. Another challenge is the difficulty in tracking the imaging location due to the absence of visual guidance during TCE imaging. While OCT images may reveal abnormalities during screening, accurate colocalization of OCT images and subsequent biopsies becomes crucial for precise diagnosis, and it is challenging to rely on the location estimated from the insertion length of the tether. Laser marking has been introduced to create visible fiducial marks in the patient’s esophagus during OCT imaging and allows precise correlation between OCT images and histopathology [34,35]. While the process requires a pause in scanning and imaging, typically for one to a few seconds, supplemental visual guidance could enhance the accuracy of positioning the capsule at the target location during marking. To maximize the clinical utilities and accelerate clinical translation, we have developed a multifunctional ablative gastrointestinal imaging capsule (MAGIC) system that can mitigate the abovementioned limitations with the traditional TCE while fully capitalizing on the benefits of the existing technology. First, the system integrates a dual-wavelength OCT imaging system operating at both 800 and 1300 nm, enabling to leverage advantages of UHR imaging of superficial layers at 800 nm and deep tissue imaging at 1300 nm. The 800-nm OCT images would allow for differentiating fine histopathological changes in mucosal layers with better resolution and enhanced contrast [31], while the 1300-nm OCT images enable monitoring the depth of lesion invasion. Second, the MAGIC includes a built-in miniature complementary metal oxide semiconductor (CMOS) camera, offering direct visualization of the esophagus in front of the capsule (similar to a conventional endoscope view) and permitting visual guidance for the capsule deployment, OCT imaging, and laser ablation. Lastly, the system is also equipped with an ablation laser, which facilitates laser marking for precise biopsies and thus can improve diagnostic yield and accuracy. By fully utilizing the critical functions in a single device, the MAGIC system can offer a comprehensive and effective solution for endoscopic screening, diagnosis, and potential ablation treatment of the esophagus.

## Results

### Super-achromatic, anastigmatic optics design for dual-wavelength OCT imaging

**Fig. 1.**
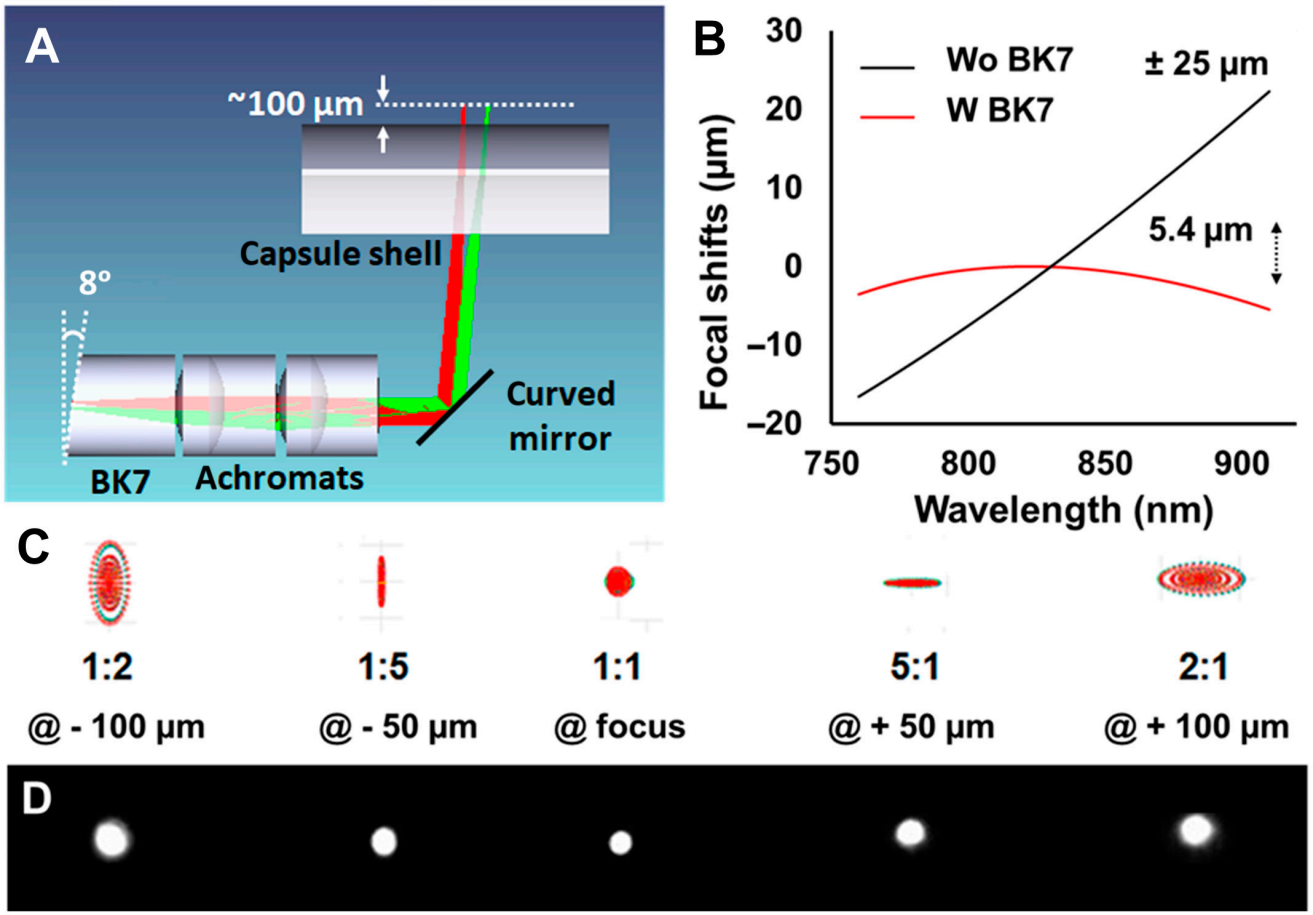
(A) Schematic of the optical layout for the MAGIC, incorporating an additional BK7 rod spacer to achieve super-achromatic performance and a curved mirror to compensate for astigmatism caused by the cylindrical capsule shell. (B) Ray-tracing results of the chromatic focal shift at a focal length of ~7 mm, with (red line) and without (black line) the BK7.Additional BK7 greatly reduced the chromatic focal shift down to ~5.4 μm, while a pair of off-the-shelf achromatic doublets alone would result in ~50-μm focal shift over the 760- to 910-nm wavelength range. (C) Ray-tracing simulation results show a pronounced astigmatism along the axial direction caused by the cylindrical capsule shell. (D) Measured output beam shape of the capsule along the axial direction where a curved mirror of a properly designed radius of curvature was used to successfully compensate the astigmatism, achieving a nearly perfect round beam shape along the axial direction.

To achieve both UHR/contrast imaging at 800 nm and deep tissue imaging at 1300 nm, we designed and optimized the optical layout of the MAGIC. Figure 1A illustrates the schematic of the optical layout. The MAGIC comprises 2 single-mode fibers with a 630-HP fiber for OCT imaging at 800 nm and an SMF-28e fiber for deep OCT imaging at 1300 nm and laser ablation at 1470 nm. The light delivered from both fibers are expanded and focused at ~100 μm outside of the capsule wall (~7 mm from the lens surface) through a super-achromatic optics assembly composed of a pair of doublets and a BK7 rod spacer. The focused imaging beams are circumferentially scanned by a curved mirror driven by a micromotor. The curved mirror had a 48° tilt angle with respect to the micromotor rotation axis in order to minimize specular reflection by the glass wall of the capsule. Two fibers are separated by 125 μm, resulting in slight differences in the focal point of ~450 and ~60 μm in the lateral and axial directions, respectively.

One of the technical challenges for achieving a UHR is to minimize chromatic aberration over a broad spectral bandwidth. It is particularly challenging at the 800-nm wavelength range due to the much stronger material dispersion than that of 1300-nm light. For example, the ray-tracing simulation results in Fig. 1B show that the combination of carefully selected achromatic doublet pair still resulted in a longitudinal chromatic focal shift of ~50 μm around the target ~7-mm focal length over the 760- to 910-nm spectral range. One method to compensate for this residual chromatic aberration is to use a custom-designed diffractive optical element [22,28]. However, it can be not only expensive for customization but also requires precise alignment along the optical axis for optimal performance. On the other hand, expanding a beam inside a dispersive material can exhibit complementary dispersion (i.e., longer wavelengths expand more than shorter wavelengths). Thus, to minimize the chromatic aberration, we introduced a BK7 rod spacer between the fiber and doublets to expand the beam, and the chromatic focal shift was further reduced by adjusting the length of the BK7 rod. At a target focal length (~7 mm), the chromatic focal shift was successfully minimized down to ~5.4 μm by using a 2-mm-long BK7 rod spacer, as shown in Fig. 1B.

Another engineering challenge for UHR imaging is astigmatism. It is known that the glass tube of the capsule functions as a negative (diverging) cylindrical lens along the azimuthal direction (perpendicular to the longitudinal axis of the capsule) and induces astigmatism [15]. Figure 1C shows the simulated beam shapes at the focus, as well as ±50 and ±100 μm away from the focal point along the imaging depth when using a conventional flat mirror on the micromotor for beam scanning. In our case, with a glass shell of a 11-mm outer diameter and a 1-mm thickness, the worst calculated astigmatism ratio (i.e., the ratio between the focused spot size in the azimuthal direction to the one in the longitudinal direction) was ~5, which would severely degrade the lateral resolution along imaging depth. To minimize this astigmatism, we thus implemented a customized curved mirror for beam scanning. The curvature of the mirror was carefully optimized to prefocus the imaging beam along the azimuthal direction, effectively compensating for the diverging effect (astigmatism) of the capsule shell. As shown in Fig. 1D, the custom curved mirror successfully alleviated astigmatism, resulting in nearly perfect round beam profiles over the entire imaging depth. The worst measured astigmatism ratio was 1.125.

### MAGIC assembly

**Fig. 2.**
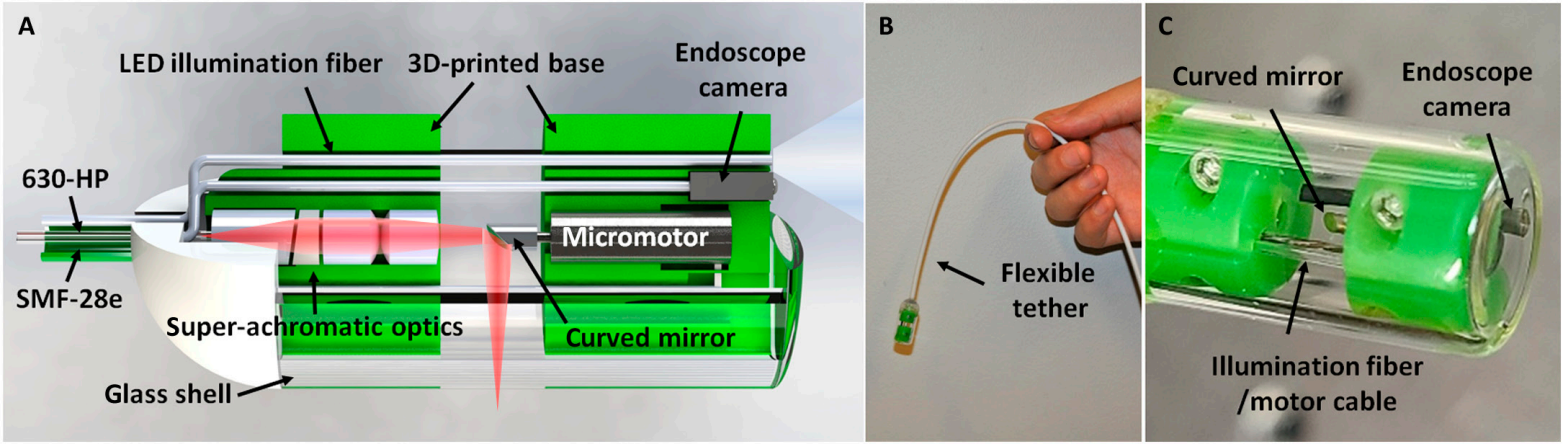
(A) Schematic illustration of the MAGIC. Two single-mode fibers for the dual-wavelength OCT imaging and laser ablation and the endoscope camera with an LED illumination fiber were all integrated inside a single device with an 11-mm diameter and a 26-mm length. (B and C) Photographs of the assembled MAGIC with a flexible tether.

Figure 2A illustrates the schematic of the MAGIC assembly. For the assembly procedure, 2 fibers of 630-HP and SMF-28e were first inserted and secured into a glass ferrule with a 2-mm diameter with epoxy. The oval-shaped bore (127 μm × 252 μm) of the ferrule accommodated both fibers. The ferrule and fiber end surfaces were angle-polished with 8° to minimize back-reflections from the fiber tips. The entry surface of the BK7 rod spacer (2 mm × 2 mm, diameter × length) that faced the fibers was also polished at an 8° angle. The angle-polished surfaces of both the ferrule and the BK7 rod spacer were aligned in parallel and then glued with an optical adhesive. This step enabled us to effectively minimize the back-reflections with a measured value of less than −55 dB.

The ferrule-spacer assembly was then carefully aligned with a pair of achromatic doublets inside a housing and mounted at the center of a base. The housing and bases were fabricated via high-precision 3-dimensional (3D) printing with a machining tolerance of better than 50 μm. The housing and base were designed to ensure concentricity between each component during assembling. The gap between the BK7 rod spacer and the achromatic lens pair was further adjusted inside the housing to precisely achieve the designed working distance of ~7 mm away from the last surface of the doublets.

The custom-designed curved mirror was securely mounted on the micromotor shaft, and then the micromotor was mounted at the center of another 3D-printed base. Two bases with the optics housing and micromotor were encased inside a glass shell enclosure consisting of a cylindrical sidewall with an 11-mm outer diameter and a flat distal end. The distance between the 2 bases was fine-tuned to position the beam focus at a desired location (100/160 μm for 800/1300 nm outside the capsule shell). An endoscope camera and an additional multimode fiber (with a 500/486-μm outer/core diameter and a numerical aperture of 0.51) for delivering a white light-emitting diode (LED) illumination light were inserted and installed in the capsule through the guiding holes on 3D-printed bases. The flat end surface of the glass housing minimizes the lensing effect and ensures a clear camera view while keeping the capsule watertight. Electrical drive wires for the micromotor and the camera and all the imaging and illumination fibers were enclosed and protected with a flexible torque coil securely connected to the proximal end of the capsule. The torque coil was further covered with a thin, biocompatible, and watertight plastic sheath suitable for in vivo use. Figure 2B shows a photograph of the assembled MAGIC with a 2-m-long flexible tether with a close-up view shown in Fig. 2C, which has a dimension of 11 mm × 26 mm (diameter × length). It is worth noting that we used a glass shell enclosure as it was readily available and had the suited size for our capsule design; however, it can ideally and conveniently be replaced with a medical-grade transparent plastic shell for future clinical applications.

### Ex vivo swine imaging

The functionality and imaging performance of the MAGIC were first demonstrated by imaging freshly dissected swine esophagi (within 30 min after sacrificing the swine). For each sample, the esophagus was first pinned on a wax block, and the capsule was gently introduced to the distal end of the esophagus following its natural path. During the deployment of the capsule, the capsule was parked at 4 random locations and ablated with the ablation laser for 20 s at 150 mW at each location. 3D volumetric OCT images of the esophagus were then acquired by pulling back the capsule with a motorized linear stage while circumferentially scanning the micromotor. Circumferential cross-sectional imaging was performed at 20 frames per second, and a total of 56-mm-long esophagus was imaged at the motorized stage pullback speed of 1 mm/s.

**Fig. 3.**
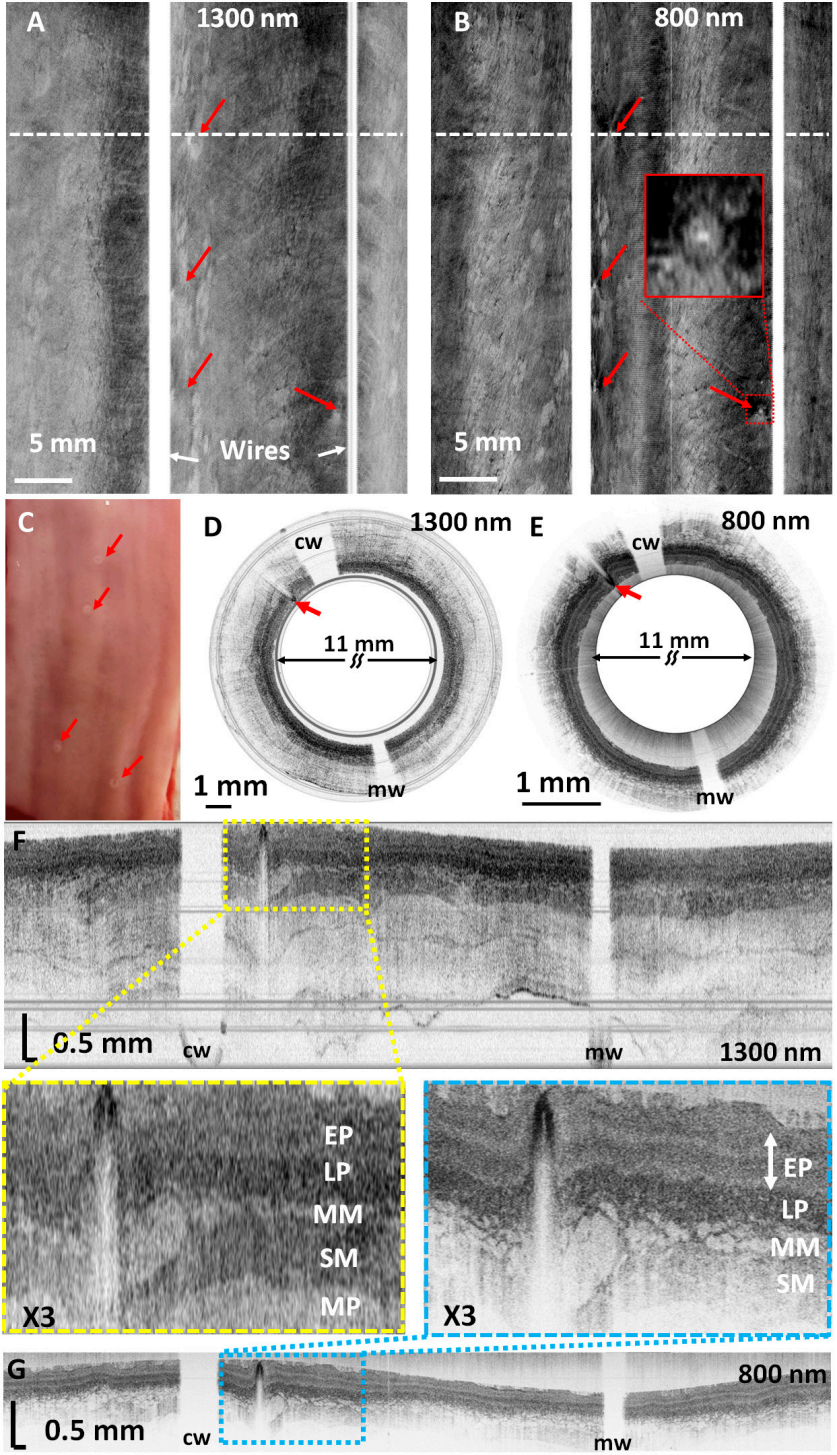
Ex vivo swine esophagus imaging results demonstrating the dual-wavelength OCT imaging capabilities and laser ablation function of the MAGIC. 3D volumetric OCT images were acquired along a 56-mm-long esophageal tract after marking 4 random spots with the ablation laser. (A and B) Unwrapped *en face* image of the 3D volumetric OCT image at 1300 nm (A) and 800 nm (B). The ablated spots are clearly visible in both images (red arrows). The red box shows an enlarged view of a representative ablated spot. (C) A photo of laser-ablated ex vivo swine esophageal tissue. The ablation spots are visible and indicated by red arrows. (D and E) Representative circumferential OCT images at an ablated spot indicated with a dashed line in (A) and (B), respectively. Red arrows indicate the ablated spot. Around 7% of the field of view was blocked by the electrical wires for the camera and the motor, visible as blank lines in (A) and (B) (white arrows), and at the 11 and 5 o’clock directions in (D) and (E) (cw, camera drive/signal wire; mw, motor drive wire). (F and G) Unwrapped rectangular images correspond to (D) and (E), respectively. 3X zoomed-in images near the ablated spot are also shown (yellow and blue dotted boxes). An OCT of 1300 nm was able to image through the esophagus layers, while 800-nm OCT only imaged through the submucosa layer but showed much detailed tissue microarchitectures, including sublayers inside the epithelium and micro-glandular structures with higher contrast. EP, stratified squamous epithelium; LP, lamina propria; MM, muscularis mucosae; SM, submucosa; MP, muscularis propria.

Figure 3A to C show *en face* images of the volumetric OCT image at both 1300 and 800 nm, and a photograph of the excised swine esophagus. The *en face* images were generated by averaging OCT signals along the depth after unwrapping each cross-sectional image. The ablation laser successfully created fiducial marks on the esophageal surface, as shown in Fig. 3C. The *en face* images provided a comprehensive overview of the entire esophagus and clearly identified the 4 marking positions, which appeared as white dots (indicated with red arrows) in both the 1300-nm (Fig. 3A) and 800-nm (Fig. 3B) OCT images. Figure 3D and E show representative circumferential (polar) cross-sectional images over one ablated spot (indicated with white dotted lines in Fig. 3A and B), which allow side-by-side comparison of the OCT imaging characteristics at 1300 and 800 nm. The unwrapped rectangular (Cartesian) images of Fig. 3D and E are also shown in Fig. 3F and G, respectively. Notably, these images do not exhibit pronounced nonuniform rotational distortions, since the micromotor was well protected within the capsule and did not experience much bending or other stresses during imaging. However, should nonuniform rotational distortions occur, several methods reported elsewhere [22,36] can be applied to effectively mitigate it.

The 1300-nm OCT revealed the full-thickness layered structures of the normal swine esophagus wall extending from the stratified squamous epithelium (EP) to lamina propria (LP), muscularis mucosa (MM), submucosa (SM), and muscularis propria (MP). Additionally, the outer wall of the esophagus could also be identified, demonstrating its capability for deep tissue imaging with an excellent resolution. In contrast, the 800-nm OCT image exhibited much finer tissue structures in the superficial layers up to the SM layer. The superb resolution and contrast of the 800-nm OCT images allow visualization of the fine tissue microstructures that are not visible in 1300-nm OCT images. Particularly, microglandular structures in the submucosal layer and the boundaries between the layers were much more pronounced compared to the 1300-nm OCT images. These performance characteristics of the 800-nm OCT would potentially enable to distinguish subtle changes in tissue microarchitectural features associated with esophageal neoplasia at an earlier stage. The current capsule design results in ~7% of image blockage due to the electrical wires for the camera and motor, as seen as blank areas in OCT images in Fig. 3. Future design would adopt thinner wires to further minimize the blockage.

### In vivo swine imaging

**Fig. 4.**
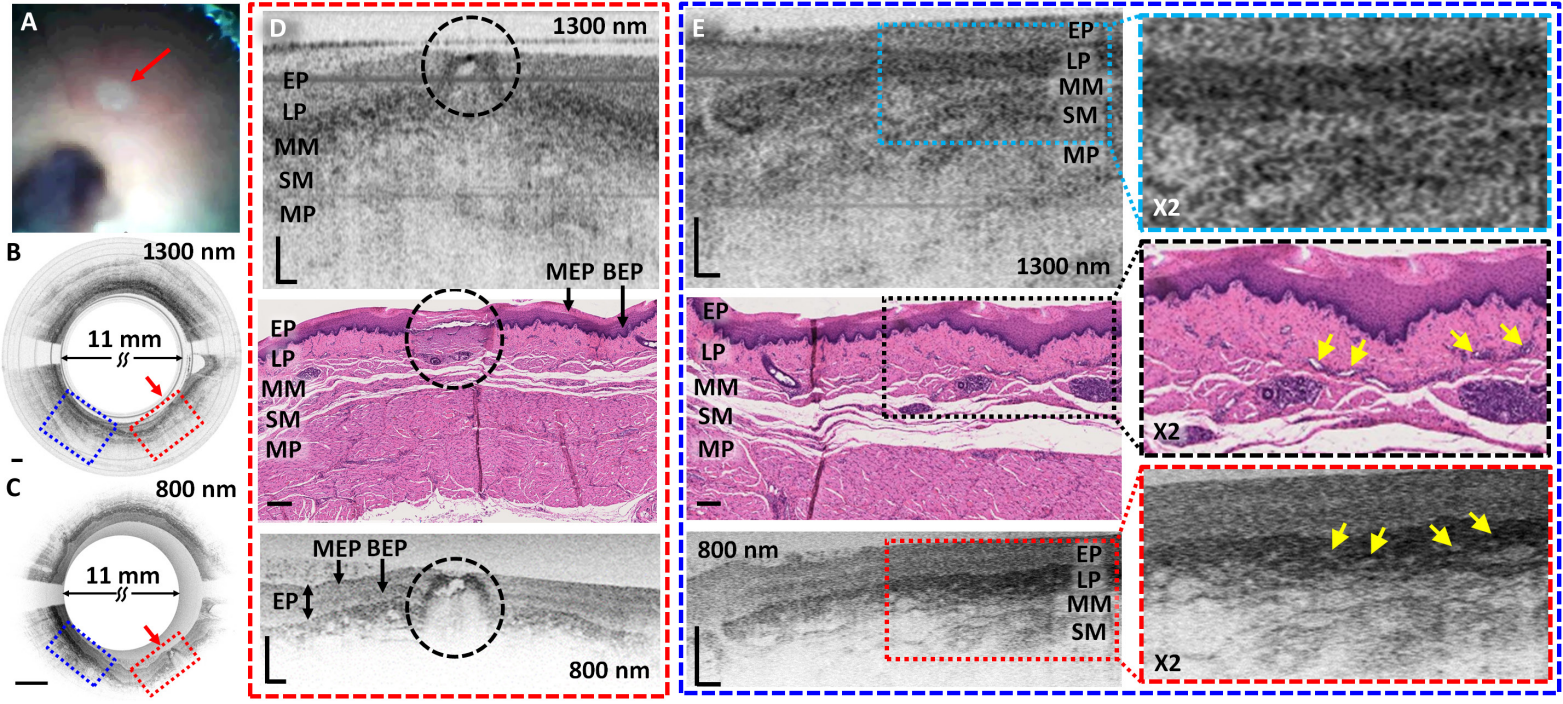
In vivo swine esophagus imaging results. (A) A representative endoscope video camera view of the esophagus. The capsule was introduced to the esophagus under the guidance of the built-in endoscope camera. (B and C) Real-time circumferential OCT images around an ablated spot captured at 1300 nm (B) and 800 nm (C). The laser-ablated spot is clearly visible in the camera image and OCT images as indicated with red arrows. (D and E) Zoomed-in OCT images and corresponding histology micrographs of the area indicated with dotted boxes in (B) and (C); red dotted box (D), and blue dotted box (E). Top and bottom images show 1300- and 800-nm OCT images, respectively, and the corresponding histology micrograph is shown in the middle. The ablated spot is indicated with dotted circles in (D). The MAGIC successfully demonstrated both deep tissue imaging at 1300 nm and UHR/contrast imaging at 800 nm. The 1300-nm OCT image revealed full-thickness layers of the swine esophagus, while the 800-nm OCT resolved micro-glandular structures buried in the subepithelium as indicated with yellow arrows in 2X zoomed-in images. EP, stratified squamous epithelium; MEP, mature epithelium; EP, basal epithelium; LP, lamina propria; MM, muscularis mucosae; SM, submucosa; MP, muscularis propria. Scale bars: 250 μm.

The performance of the MAGIC was further demonstrated by swine esophagus imaging in vivo. The capsule was introduced into the esophagus of a sedated swine under the visual guidance of the built-in camera and the real-time feedback from the simultaneous 800 and 1300-nm OCT imaging. During the capsule insertion, random locations were selected to simulate “suspected” areas and ablated for 3 s to create visible fiducial marks. These marks serve not as indicators of abnormality but as references for potential areas of interest. In clinical scenarios, upon detecting a genuinely abnormal area in the OCT images or video footage, defined by characteristics suggesting pathology or concern, the MAGIC allows for these specific areas to be ablated, creating a reference mark for further examination or treatment. The consistency of the laser marking may be affected by unstable contact of the capsule with the esophageal wall due to the peristalsis. To mitigate this issue, we utilized a high-power pulsed Raman laser, which has demonstrated the capability of making effective marks and minimizing the impact of transient tissue contact [35,37]. In practice, the MAGIC allows immediate verification of tissue coagulation at the targeted area through subsequent OCT scans. The MAGIC is designed to ensure that the locations of the laser marks correspond precisely with the areas being imaged by sharing the same optics. If the subsequent OCT scan indicates that the initial marking was insufficient, a prompt remarking can be performed. As shown in Fig. 4A, the ablated spots can be clearly visible in the endoscope camera video footage, allowing for accurate correlation during further examination or other procedures. Subsequently, the marked areas were imaged again with the capsule (Fig. 4B and C) and biopsied at the end of the study. The Movie S1 shows real-time OCT images at 1300 and 800 nm, along with the endoscope camera video footage captured simultaneously by the MAGIC. The OCT images were compared with the corresponding histology micrographs of the biopsied tissue samples. Figure 4D to E show zoomed-in OCT images and the corresponding histology micrographs near the ablated spot (dotted red and blue box in Fig. 4B and C).

The ablated tissue site could be easily identified in both OCT images and the histology micrograph with good correlation (indicated with black circles in Fig. 4D). The histology micrograph confirmed that the lateral extent of the thermal damaged area was ~700 μm with the depth reaching ~500 μm, extending to the boundary of the MM layer while creating a cavity in the epithelial layer (where the beam was focused). These details were also clearly identifiable in the OCT images, suggesting its capability to detect and visualize microarchitectural changes and monitor the depth of invasion. The dual-wavelength OCT imaging enables UHR/contrast imaging of superficial layers as well as deep tissue layers. As shown in Fig. 4E, the 1300-nm OCT enabled to image entire tissue layers of the swine esophagus. At the same time, the 800-nm OCT could resolve the microglandular structures underneath the squamous epithelium with much improved resolution and contrast. The 2X zoomed-in images of Fig. 4E clearly identify the fine details of the microglands or ducts, as indicated with yellow arrows, suggesting that UHR imaging at 800 nm could play a crucial role in assessing Barrett’s glands buried underneath the subepithelium [27,38] and for achieving curative treatment. Notably, the 800-nm OCT images reveal sublayers in the epithelium, as seen in the color gradient in histology images. This may represent the development of the epithelium layer from the basal layer with stem cells toward matured superficial layers [39,40]. Conventional eOCT at 1300 nm has shown the potential to identify irregular glands. However, it remains challenging to identify dysplastic glands or changes due to its insufficient resolution and contrast [25–27]. The 800-nm OCT image in Fig. 4E shows finer microstructures of the subepithelial glands, which cannot be clearly resolved in the 1300-nm images. The UHR imaging capability of the MAGIC holds strong promise to augment clinical diagnosis and treatment assessment.

## Discussion

The MAGIC capable of dual-wavelength OCT imaging, laser ablation, and endoscope camera imaging was developed, and its functionalities were successfully demonstrated in ex vivo and in vivo swine model imaging studies. With dual-wavelength OCT imaging, operating at 800 and 1300 nm simultaneously, the MAGIC provides high-resolution images from the superficial mucosal layers to deeper tissue layers. The UHR OCT images at 800 nm offer enhanced visualization of fine tissue microarchitecture, potentially allowing for the detection of subtle changes associated with early esophageal neoplasia, while 1300-nm OCT imaging provides deeper penetration, which is crucial for evaluating lesion invasion (highly relevant to selecting the proper treatment). Furthermore, the ablation laser enabled precise marking of suspicious areas, facilitating subsequent targeted biopsies and improving diagnostic accuracy. Although the therapeutic potential of laser ablation was not specifically demonstrated in this study, it holds promise for ablative treatment of esophageal lesions, particularly early-stage lesions. The MAGIC could facilitate precise identification of lesions through high-resolution OCT imaging. Subsequent treatment with the integrated ablation laser could be conducted, selectively targeting well-defined areas of abnormal superficial tissue and minimizing damage to the adjacent or deeper healthy tissues. Unlike traditional fiber-optic-based laser ablation, which frequently demands multiple sessions [41], the MAGIC provides 3D scanning capabilities (circumferential and pullback). It could facilitate the ablation of as extensive area akin to conventional radiofrequency ablation, while the depth of tissue ablation can be more precisely controlled [42]. This feature would allow transforming the MAGIC into a theragnostic device capable of both diagnosis and treatment within a single procedure. Lastly, the endoscope camera integrated within the MAGIC further enhances its functionality. The current MAGIC design utilizes a low-resolution (400 × 400 pixels) camera that is limited for diagnostic purposes. Future adoption of a medical-grade, high-resolution endoscope camera would maximize its clinical utility. The endoscope camera affords comprehensive tissue surface surveillance along the GI tract, enabling initial screening and providing real-time visual guidance for navigating and positioning the capsule. In the current design of our system, the endoscope camera captures video in front of the capsule, making it challenging to achieve exact correlation with OCT images. Despite this, the distance between the location on the camera view and the OCT view in principle can be roughly estimated. If an abnormal area is detected in the video footage, it would be very likely identifiable in the preceding OCT cross-sectional or *en face* images (assuming imaging is performed during the insertion of the capsule within a lumen). Moreover, the MAGIC system is designed to facilitate comprehensive scanning of the esophagus from the gastroesophageal junction to the mouth, offering a potential means to correlate recorded endoscopic and OCT images with specific locations along the tract and further registering with other imaging modalities [43]. A more accurate correlation would be possible with the help of the laser ablation landmarks during the following rounds of imaging. The camera images would also offer clinicians familiar functionality similar to conventional GI endoscopic procedures. It would not only facilitate the interpretation and analysis of OCT images but also contribute to accelerating the clinical translation and adaption of the TCE technology. Moreover, the MAGIC affords more advanced endoscopic imaging functions by utilizing red, green, and blue (RGB) laser illumination instead of LED [44]. This opens up possibilities for applications for narrow-band imaging [45] and photodynamic therapy [46] (e.g., using 5-aminolevulinic acid [47] or porfimer sodium [48]), allowing for versatile imaging and treatment using a single device.

The MAGIC successfully integrates clinically viable functions for comprehensive esophagus surveillance and potential ablation treatment with surface and volumetric imaging guidance and feedback. This advancement holds a strong promise in accelerating the clinical translation and adoption of the TCE technology. This study utilized 2 separate systems (as described in Dual-wavelength OCT imaging system) for each wavelength to highlight their distinct advantages and synergistic, complementary benefits when combined. Our results suggest that integrating these 2 wavelengths into a single device enables more comprehensive diagnosis, potentially enhancing clinical outcomes. In light of the practical considerations for clinical application, it is essential to develop a singular, portable, integrated system once proven clinically needed, while it is feasible by leveraging compact fiber-optic components and turn-key solutions that are currently available for both 800- and 1300-nm wavelengths. Efforts are underway to continue the development and conduct pilot clinical validation studies, aiming to optimize and unleash the clinical potential of the technology.

## Materials and Methods

### Dual-wavelength OCT imaging system

**Fig. 5.**
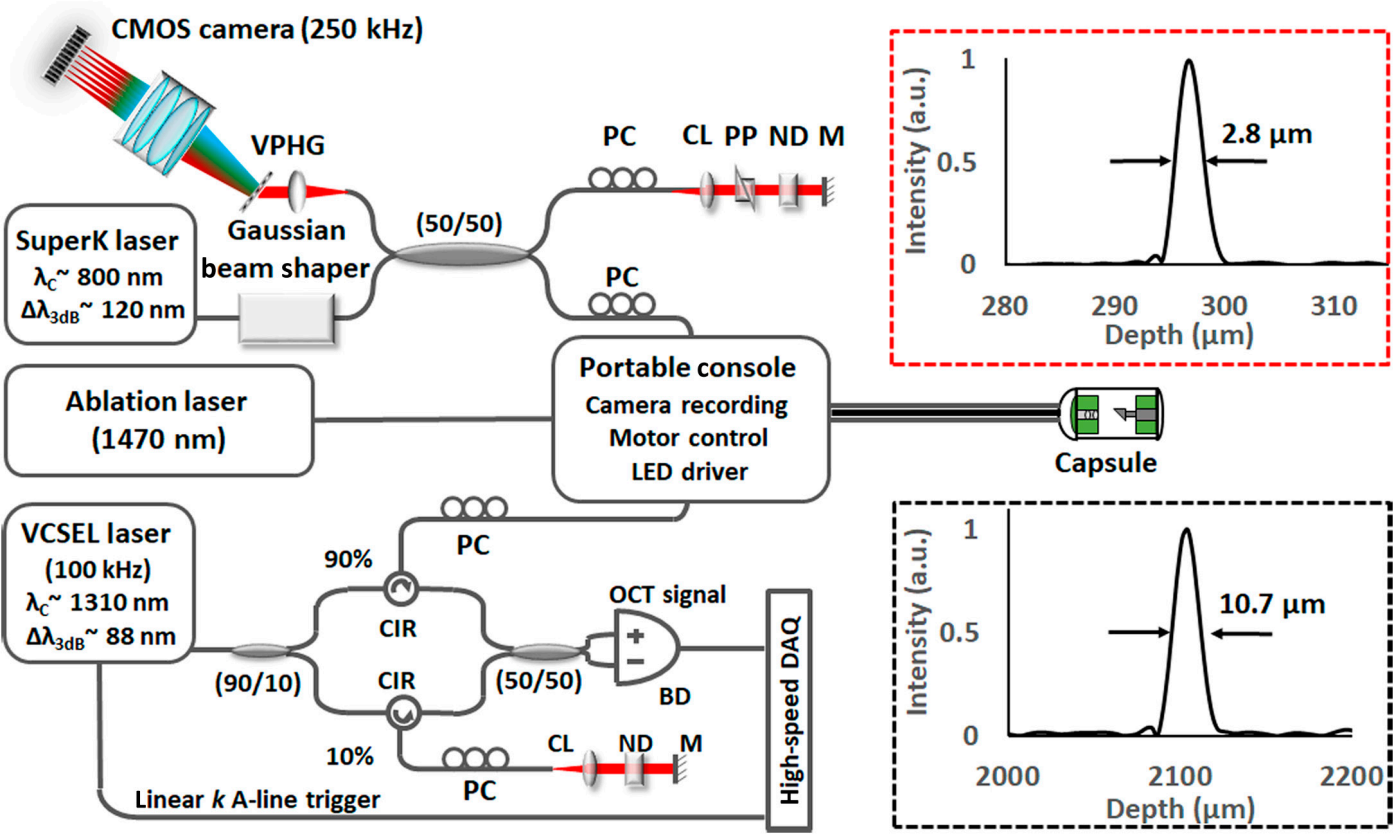
Schematic of the MAGIC system consists of a dual-wavelength OCT imaging system along with laser ablation and an endoscopic video camera. All the fiber-coupled light sources, including OCT imaging beams at both 800 and 1300 nm, ablation laser, and illumination LED, are delivered to the capsule and controlled through a portable console. The console also includes an endoscope camera recording system, a micromotor controller, and an LED driver. The red and black dashed boxes show the measured axial point spread functions (PSFs) of the capsule when using the 800- and 1300-nm OCT imaging systems, respectively, with the axial resolution given by the full-width-at-half-maximum of the corresponding PSF. CL, collimating lens; ND, neutral density filter; M, mirror; PC, polarization controller; PP, prism pair; VPHG, volume phase holographic grating; CIR, fiber-optic circulator; BD, balanced detector.

Figure 5 shows the schematic of the MAGIC imaging system. It consists of 3 independent portable systems: an UHR spectral-domain OCT (SD-OCT) system operating at 800 nm, a deep tissue imaging swept-source OCT (SS-OCT) system operating at 1300 nm, and an ultracompact endoscope video camera system. An ablation laser at 1470 nm was also integrated into the system. All systems were integrated and controlled through a portable console. The console includes microcontrollers for the micromotor and the camera, an LED light source and its controller, and fiber connectors to transceive light between each imaging system and the capsule. The output powers of the OCT imaging beams at the capsule were adjusted to ~16 mW for 800 nm and ~7 mW for 1300 nm.

The UHR SD-OCT system utilizes a supercontinuum (SuperK) light source (NKT Photonics) equipped with a Gaussian beam shaper, which provides a 3-dB spectral bandwidth of ~120 nm centered at ~800 nm. The laser was coupled in a fiber-optic Michaelson interferometer and separated into the sample and reference arms using a 50/50 broadband fiber-optic coupler. The OCT interference signal was captured by a spectrometer with a line-CMOS camera (Wasatch Photonics) that can operate at up to 250-kHz A-line scan rate. The spectrometer was designed to accommodate a spectral bandwidth of 300 nm with a spectral resolution of ~0.15 nm/pixel, enabling a calibrated imaging depth of 1 mm. With super-achromatic optics described in the previous section, an ultrahigh axial resolution of 2.8 μm in air was achieved (red dotted box in Fig. 5).

The deep-tissue imaging SS-OCT imaging system consists of a vertical-cavity surface-emitting laser (VCSEL) swept laser (Excelitas Technologies) and a fiber-optic Mach-Zehnder interferometer. The VCSEL provided a 100-kHz A-line scan rate at a central wavelength of 1310 nm with a 3-dB spectral bandwidth of ~88 nm (and a 10-dB bandwidth of ~130 nm). The laser was split into the sample and reference arms using a 90/10 fiber-optic coupler, and the OCT interference signal was obtained through a balanced detector. The VCSEL source incorporated an internal linear *k*-clock (with an imaging depth of up to 6 mm in air) to trigger a high-speed DAQ card (AlazarTech) for synchronized data acquisition. The measured axial resolution of the SS-OCT system was ~10.7 μm in air (black dotted box in Fig. 5).

### Endoscope video camera system

The video camera system comprises a compact CMOS camera (1.05 mm × 1.05 mm × 2.27 mm) and a fiber-coupled white LED (Thorlabs, MCWHF2) for illumination. The ultracompact CMOS sensor, along with optical lenses (OVM 6946, OmniVision) provides a 120° wide field of view with a focusing range of 5 to 50 mm. The camera captures images of 400 × 400 pixels at 4.33 frames per second.

### Laser ablation system

Two different types of ablation lasers were used in the studies. A fiber-coupled diode laser (Anritsu, 500 mW) was first used for ex vivo studies. This laser was later replaced by a pulsed Raman fiber laser (IPG Photonics, 3W) for in vivo studies, which was more efficient and required a much shorter time to ablate the tissue with much reduced motion artifacts in the in vivo environment. Both lasers operate at 1470 nm and can be directly coupled into the SMF-28e fiber and focused on the target tissue surface through the same capsule optics.

### Animal and study approval

All animal housing and experiments were performed under the guidelines described in the National Institutes of Health Guide for the Care and Use of Laboratory Animals. The protocol was approved by the institutional animal care and use committee of the Johns Hopkins University.

### In vivo swine imaging protocol

To ensure the safe passage of the capsule into the esophagus, an overtube with an inner diameter slightly larger than the capsule was employed. The capsule was directly introduced into the overtube, and then the overtube was slowly retracted. Subsequently, the capsule was further inserted toward the gastroesophageal junction under the guidance of the built-in endoscope camera and real-time OCT imaging. Real-time OCT images of the esophagus could be acquired during either manual pullback or insertion of the capsule. A pulsed Raman fiber laser was employed with a power higher than the one used for the ex vivo studies in order to effectively create fiducial marks within a short period of time (3 s). The output power of the fiber laser was adjusted to 850 mW (at 1470 nm) and operated at 10 kHz.

## Data Availability

All data needed to evaluate the conclusions in the paper are present in the paper and/or the Supplementary Materials. Additional data related to this paper may be requested from the corresponding author.
